# Health-Related Quality of Life and Associated Factors Among Oldest-Old in China

**DOI:** 10.1007/s12603-020-1327-2

**Published:** 2020-01-20

**Authors:** C. Chen, G. G. Liu, Q. L. Shi, Y. Sun, H. Zhang, M. J. Wang, H. P. Jia, Y. L. Zhao, Yao Yao

**Affiliations:** 1 National School of Development, Peking University, 5 Yiheyuan Road, Haidian District, Beijing, China; 2 Department of Symptom Research, The University of Texas, MD Anderson Cancer Center, Houston, TX USA; 3 China Center for Health Economic Research, Peking University, Beijing, China; 4 Cornell University, Ithaca, NY USA; 5 Central Laboratory, Hainan Hospital of Chinese PLA General Hospital, Sanya, Hainan, China

**Keywords:** EQ-5D-3L, oldest old, lifestyle, social and family support, China

## Abstract

**Objectives:**

The oldest old population has become the fastest growing segment with excess need of care and social support, it is crucial to improve the health-related quality of life (HRQoL) of these populations. This study seeks to evaluate the health status and to investigate modifiable factors associated with health-related quality of life for oldest old adults in China.

**Design:**

A cross-sectional population-based study.

**Setting:**

Hainan Province in the south of China.

**Participants:**

1,278 adults aged 80 years or older.

**Methods:**

HRQoL was assessed by three-level EuroQol-5D scale (EQ-5D-3L) and a visual analogue scale (VAS). Demographic and health-related variables were analysed by estimating mean values and standard deviations for continuous variables, percentages and standard deviations for categorical variables. Tobit regressions, ordinary least Squared (OLS) regressions and ordered probit regressions were adopted to determine the associated factors for overall HRQoL and for each health dimension.

**Results:**

Anxiety/depression was the least reported problem while mobility was the most frequently reported with problem. Female respondents had lower EQ-5D score (0.76 vs. 0.86) and VAS score (66.55 vs. 69.84) than male respondents. Better health-related quality of life was significantly associated with higher BMI, no drinking habit, more leisure activities, living with family members, good sleeping quality, closer social and family connections, fewer numbers of drugs consumed per day, without having hearing or visual impairment, and fewer chronic conditions, after controlling for potential confounders.

**Conclusion:**

Findings from this study suggested that quality of life was not only associated with age-related diseases, but also correlated with a range of health-related lifestyles, and factors indicating social and family support.

**Electronic Supplementary Material:**

Supplementary material is available for this article at 10.1007/s12603-020-1327-2 and is accessible for authorized users.

## Introduction

The proportion of oldest old population (aged 80 and older) has increased from 0.95% in 2000 to 1.57% in 2010 according to the Population Census in China (1). The oldest old population has become the fastest growing segment under the steady economic development and improved health services. Several challenges come with this demographic pattern and need to be addressed to improve the health-related quality of life of the oldest old adults (2). This study seeks to evaluate their health status and to investigate modifiable factors associated with health-related quality of life to initiate appropriate public health policies.

In this study, we used the EuroQol-5D (EQ-5D) instrument to measure health-related quality of life for the oldest-old adults in China. The EQ-5D instrument has been utilized in various contexts, including outcomes in clinical practices, evaluations in health economic research or utility index in healthcare interventions (3). There is also a growing interest in applying EQ-5D to the measurement of population health and the results show evidence for its validity (4–7). Yang et al. developed population norms for the EQ-5D dimensions based upon a representative sample of urban Chinese (5). Yet few studies have used EQ-5D to investigate the prevalence and determinants of the health-related quality of life for oldest old adults, especially in Asian countries such as China. Studies from other countries indicated that age, gender, education level, comorbidities, living arrangements and social support were important determinants for health among the elderly population (8–11).

Healthy aging is a critical aspect of wellbeing among elders and a fundamental objective of the recent government institutional reform in China (Establishment of Department of Aging Health in 2018). Accordingly, an examination of health-related quality of life and associated factors is vital for health policymakers. The information may provide some implications for developing effective public health strategies to maintain a healthy life of high quality among oldest-old adults. In this study, we aim to address the following questions by using a cohort aged 80 years and over in China: 1) Compared with western settings, how well is the health quality of life of the oldest-old population in China? 2) Which modifiable factors may have impacts and interventional potential on the health-related quality of life among this population?

## Methods

### Study design and study population

The sample of this study was obtained from the first wave of China Hainan Centenarian Cohort Study (CHCCS). Conducted from 2014 to 2016 in Hainan province, it was one of the largest oldest-old adults’ health interdisciplinary studies in China. Hainan province, the southernmost province of mainland China, was observed with one of the highest life expectancies (76.3 years in 2010 national census) and the highest percentage of centenarians (18.75/100000 in 2014) in China (12).

The survey included a representative sample of the individuals aged between 80–99 years old and centenarians (≥100 years old) in Hainan Province. Details of the sampling strategy for centenarians were described in other studies (13, 14). For the individuals aged between 80–99, stratified random sampling was adopted. Based on the National Civil Registry, residents aged between 80–99 years old were stratified by gender, age, geographic location, and population density to ensure the representativeness. The survey was conducted among all centenarians with the help of the residence list provided by Hainan Provincial Civil department. Face-to-face questionnaire interview, clinical examination, and biological specimens collecting were implemented for each participant to collect information related to their socio-economic conditions, physical and mental health status. A total of 1,278 adults aged 80 years and older were included in the final analyses.

All participants were informed about the research contents and signed consent forms before completing the questionnaire interview, physical health examination, and blood tests. This study was approved by the Ethics Committee of the Hainan Branch of Chinese People’s Liberation Army General Hospital (301hn11201601).

### Health status using the EQ-5D-3L instrument

The three-level EuroQol-5D scale (EQ-5D-3L) were used in the face-to-face interviews to measure the health-related quality of life of the oldest-old respondents. The EQ-5D-3L instrument is widely used to evaluate an individual’s overall health status using a description system and a visual analogue scale (VAS) (15). The description system consists of five questions on five dimensions (5D): mobility, self-care, usual activities, pain/discomfort, and anxiety/depression (16). Mobility dimension asks about the person’s walking ability. Self-care dimension asks about the ability to wash or dress by oneself, and usual activities dimension measures performance in work, study, housework, family or leisure activities. In pain/discomfort dimension, it asks how much pain or discomfort they have, and in anxiety/depression dimension, it asks how anxious or depressed they are. Each dimension is measured by three-level of severity, including no problems, some/moderate problems, and severe/extreme problems. The respondents were inquired to self-rate their health status in each of the five dimensions. The visual analogue scale (EQ-VAS) score records an individual’s self-rated health on a 20 cm, vertical visual analogue scale ranging from 0–100, with notes at both ends labelling “the worst health you can imagine” (at 0) and “the best health you can imagine” (at 100). The Chinese version of the EQ-5D-3L questionnaire were used in this study (17).

According to respondents’ EQ-5D answers, a combined health utility score was calculated with 1 for full health and 0 for death. The responses to 5 health dimensions were weighted using a study on Chinese general population preferences-the Chinese version of time trade-off value set (TTO) (Supplement Table 1)—for each health dimension (18). The weights represent differences in 3 severity levels associated with each health dimension. An N3 (0.022 in Chinese TTO) would be deducted in the algorithm when there was at least one “severe problem” reported in the answer. The EQ-5D-3L N3 term is an extra weight to deduct when at least one of the dimensions is at the most severe level, and thus this further reduces the overall EQ-5D score. For example, the EQ-5D score from the answer of “12113” would be calculated as: 1-0-0.105-0-0-0.205-0.022

**Table 1:** General Characteristics of the Participants

	**Whole Sample (n=1278)**	**Male(n=402)**	**Female(n=876)**	**X**^**2**^**or t (P-value)**
Age (years)	93.65±9.57	90.13±9.12	95.26±9.34	9.197(<0.001)
Illiterate	1058(82.79)	231(57.46)	827(94.41)	263.897(<0.001)
Married (spouse alive)	415(32.47)	213(52.99)	202(23.06)	112.534(<0.001)
Widowed	848(66.35)	179(44.53)	669(76.37)	125.144(<0.001)
Chronic conditions	400(31.30)	129(32.09)	271(30.94)	0.174(0.917)
No. of drugs	0.42±0.82	0.49±0.89	0.39±0.79	1.818(0.693)
Hearing impairment	218(17.06)	44(10.95)	174(19.86)	15.489(<0.001)
Visual impairment	265(20.74)	82(20.40)	183(20.89)	0.401(0.840)
BMI	19.62±3.83	20.60±3.46	19.17±3.91	6.290(<0.001)
Underweight	503(39.36)	102(25.37)	401(45.78)	48.060(<0.001)
Normal weight	624(48.83)	235(58.46)	389(44.41)	21.774(<0.001)
Overweight	123(9.62)	58(14.43)	65(7.42)	15.557(<0.001)
Obese	28(2.19)	7(1.74)	21(2.40)	0.553(0.457)
Smoking	95(7.43)	78(19.40)	17(1.94)	122.111(<0.001)
Drinking	178(13.93)	104(25.87)	74(8.45)	69.775(<0.001)
Good sleeping quality	467(36.54)	160(39.80)	307(35.05)	2.687(0.101)
Normal sleep quality	684(53.52)	205(51.00)	479(54.68)	1.504(0.220)
Poor sleep quality	127(9.94)	37(9.20)	90(10.27)	0.353(0.553)
No. of Leisure activity	1.25±1.01	1.66±1.06	1.06±0.92	10.244(<0.001)
Living alone	231(18.08)	88(21.89)	143(16.32)	5.766(0.016)
Friends connection	540(42.25)	186(46.27)	354(40.41)	3.875(0.049)
Family connection	1199(93.82)	379(94.28)	820(93.61)	0.214 (0.644)

### Demographic and health-related variables

Demographics including age, gender, level of education, and marital status were collected according to the structured questionnaire. The home interview also collected information on number of drugs consumed per day, self-rated sleeping quality, types of leisure activities, drinking and smoking behaviour, hearing impairment and visual impairment, whether the respondent had steady friends and family members to contact and seek for help every month, and whether the respondent was living alone.

Health examinations were performed to collect health-related information. Height and weight were measured by standard procedures. Body mass index (BMI) was calculated by dividing weight in kilograms by the height in metres squared. We further divided the BMI into underweight (<18.5 kg/m^2^), normal weight (18.5–24 kg/m^2^), overweight (24–28/kg/m^2^), and obesity (≥28 kg/m^2^) following the World Health Organization (WHO) criteria (19). The respondent was recorded as having chronic diseases if she or he had at least one of the following conditions: cardiovascular disease, hypertension, diabetes, and hyperlipidaemia and cancer. Cancer was self-reported if the respondent was diagnosed by doctors. The rest of chronic diseases were obtained by clinical health examination. Systolic blood pressure (SBP) and diastolic blood pressure (DBP) were reported as the average of two measurements, which were conducted consecutively with an at least 1-minute interval in between. Respondents who had SBP ≥ 140 mmHg or DBP≥ 90 mmHg or those taking antihypertensive medication were recorded as hypertensive (20). The venous blood sample was taken when respondents were in a seated position and was transported in cold storage (4°C) to the Central Laboratory within 4 hours. Fasting blood glucose (FBG), serum concentrations of triglyceride (TG), total cholesterol (TC), low-density lipoprotein cholesterol (LDL-c), and high-density lipoprotein cholesterol (HDL-c) were measured accordingly. The respondents who had FBG ≥7.0 mmol/L or those with antidiabetic medication were reported to have diabetes. The respondents who had TG ≥1.7 mmol/L, TC ≥5.18 mmol/L, LDL-c ≥3.37 mmol/L, HDL-c <1.04 mmol/L or who were treated with lipid-regulating medication were considered to have dyslipidaemia.

### Statistical Analysis

Demographic and health-related variables were analysed by estimating mean values and standard deviations for continuous variables, percentages and standard deviations for categorical variables. We also described the distribution of respondents who answered the questions of EQ-5D descriptive system and EQ-VAS. The results were presented by gender and as a whole sample. Differences between gender-groups were tested using t-tests for continuous variables and chi-squared tests for categorial variables.

Tobit regressions were employed to estimate the association between relevant factors and EQ-5D scores since the EQ-5D score (dependent variable) is censored at 1 (full health). Ordinary Least Squared (OLS) regressions were used to quantify the effects of risk factors on the EQ-VAS score.

Given the Tobit and OLS models identify the risk factors for the combined health quality (EQ-5D and VAS scores), ordered probit regressions were adopted to determine the associated factors for each health dimension. The dependent variables were a discreet set of outcomes with the natural order, including no problems, moderate problems, and severe problems. All the analyses were carried out by using Stata 14.0 Versions (21).

## Results

### Demographic and health-related characteristics

Table 1 presented the general and gender-specific characteristics of the respondents. Among the 1,278 respondents, 402 were male and 876 were female, with a total mean age of 93.65 years. Men on average were 5 years younger than women (p<0.001). More than half of the men (57%) and 94% of the women were illiterate. About one-quarter of the women (23%) were married and nearly three-quarters of them (76%) were widowed. Men had a higher proportion of being married (53%) and a lower (45%) percentage of being widowed (*p* <0.001). Around 30% of the sample had at least one chronic disease, while 40% of them didn’t answer the question. They consumed less than 1 type of drugs per day. Approximately 20% of the respondents had hearing or visual impairment. No significant gender differences were observed in terms of disease related variables. The overall sample had an average BMI of 19.62, among which more women (46%) were underweight than men (25%). Nearly half of the respondents had normal weight, while less than 10% of the women were overweight or being obese. Smoking and drinking are more prevalent in men than in women despite only less than 20% of the whole sample smoke and 26% of the sample consume alcohol. About 90% of the respondents had normal or good sleeping quality. The respondents at least had 1.25 types of leisure activities on average. More men (22%) were reported to live alone than women (16%), while 42% of the respondents had social contacts and 94% of them had family contacts every month.

### EQ-5D and EQ-VAS score

The distributions of 5 health dimensions, EQ-5D score, and EQ-VAS score were presented in Table 2. The average EQ-5D score was 0.79 while the VAS score was 66.90 for the whole sample. Among 5 health dimensions, anxiety/depression was the least reported problem (12%), while mobility was the most frequently reported with complaint (50%). Approximately one third of the oldest-old respondents reported no problems in all health dimensions, while 6.3% reported having problems in all five dimensions.

**Table 2:** Frequency Distribution of replies in the EuroQol 5D scale

**EuroQol 5D scale**		**Whole sample (n=1278)**	**Male (n=402)**	**Female (n=876)**	**X**^**2**^**or t (P-value)**
Mobility (n, %)					63.369(<0.001)
	No problems	638(49.92)	265(65.92)	373(42.58)	
	Moderate problems	596(46.64)	133(33.08)	463(52.85)	
	Extreme problems	44(3.44)	4(1.00)	40(4.57)	
Self-care (n, %)					34.097(<0.001)
	No problems	829(64.87)	305(75.87)	524(59.82)	
	Moderate problems	408(31.92)	93(23.13)	315(35.96)	
	Extreme problems	41(3.21)	4(1.00)	37(4.22)	
Usual activities (n, %)					47.729(<0.001)
	No problems	657(51.41)	263(65.42)	394(44.98)	
	Moderate problems	572(44.76)	132(32.84)	440(50.23)	
	Extreme problems	49(3.83)	7(1.74)	42(4.79)	
Pain/discomfort (n, %)					21.635(<0.001)
	No problems	800(62.60)	289(71.89)	511(58.33)	
	Moderate problems	474(37.09)	112(27.86)	362(41.32)	
	Extreme problems	4(0.31)	1(0.25)	3(0.34)	
Anxiety/depression (n, %)					4.935(0.085)
	No problems	1119(87.56)	364(90.55)	755(86.19)	
	Moderate problems	156(12.21)	37(9.20)	119(13.58)	
	Extreme problems	3(0.23)	1(0.25)	2(0.23)	
Any problems in EQ-5D domains (n, %)					66.009(<0.001)
	No problems	452(35.37)	202(50.25)	250(28.54)	
	1 domain	174(13.62)	58(14.43)	116(13.24)	
	2 domains	151(11.82)	37(9.20)	114(13.01)	
	3 domains	213(16.67)	45(11.19)	168(19.18)	
	4 domains	208(16.28)	43(10.70)	165(18.84)	
	5 domains	80(6.26)	17(4.23)	63(7.19)	
EQ-5D-3L score (mean±sd)		0.79±0.2	0.86±0.2	0.76±0.2	7.972(<0.001)
EQ-VAS score (mean±sd)		66.90±12.9	69.84±12.1	65.55±13.0	5.595(<0.001)

We found significant differences between male and female respondents in every health dimension, except for anxiety/depression. Approximately half of the men (50%) reported no problems in any health domains, while only 29% of the women reported no problems. Accordingly, female respondents had lower EQ-5D score (0.76) and VAS score (66.55) than male respondents (0.86 & 69.84) with the p-values<0.001.

**Figure 1:**
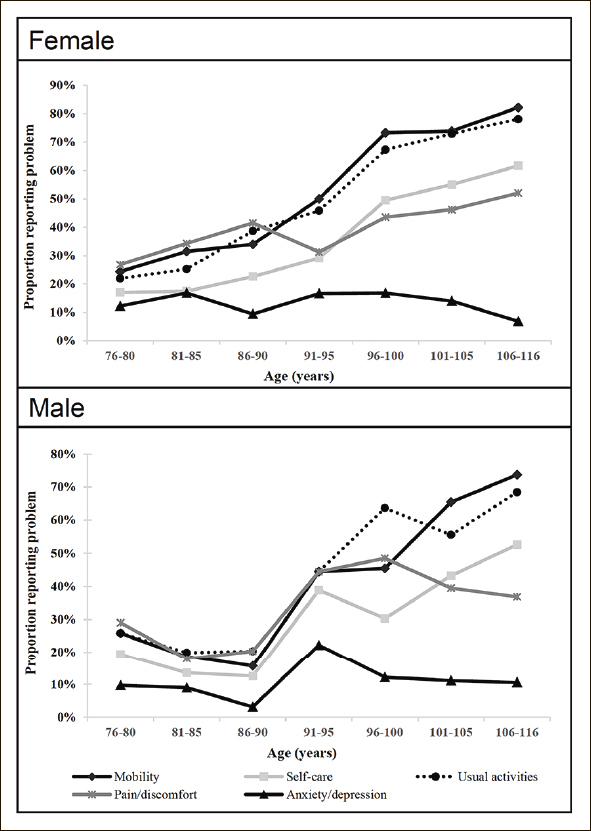
Percentage of reporting any problems in 5 health domains

Figure 1 showed the patterns of proportions reporting problems in 5 health dimensions across different age groups. The upper panel presented the trends for female respondents, while the lower panel was for male respondents. In general, there was an increasing trend of reporting problems as the respondents getting older in all dimensions except for anxiety/depression. For male respondents, there was a sharp increase in the age groups of 86 to 95 for all health dimensions. After that, there were fewer problems reported in the dimension of anxiety/ depression. This sharp increase was not observed among female respondents. Once female respondents reached 91 years old, they were less likely to report problems in anxiety/depression.

### Associated factors of combined health utility

Table 3 presented the results on the EQ-5D score from Tobit regressions and EQ-VAS score from OLS regressions. The associated factors of both scores were generally consistent, including age, visual impairment, BMI, sleeping quality, number of leisure type, and living alone. While gender, drinking behaviour, number of drugs consumed per day, hearing impairment were only correlated to EQ-5D score, but not significantly associated with EQ-VAS score. Chronic diseases and having social contact significantly affect EQ-VAS score rather than EQ-5D score.

**Table 3:** Regression Analyses on QALY and EQ-VAS Score

	**Regression coefficients (p-value)**	
	**EQ-5D score**	**EQ-VAS score**
Age	−0.005*** (<0.001)	−0.168*** (<0.001)
Male	0.032 (0.082)	0.735 (0.399)
Illiterate	0.029 (0.201)	−0.990 (0.320)
Married	0.005 (0.949)	0.441 (0.885)
Widowed	−0.096 (0.238)	−0.296 (0.922)
Chronic diseases (no combabilities=reference)	−0.007 (0.697)	−1.601 (0.082)
No. of drugs consumed per day	−0.032*** (<0.001)	−0.430 (0.331)
Hearing impairment	−0.084*** (<0.001)	−0.396 (0.678)
Visual impairment	−0.048** (0.004)	−2.122* (0.017)
BMI	0.004* (0.041)	0.161 (0.089)
Smoking	0.034 (0.287)	−0.087 (0.947)
Drinking	−0.059** (0.002)	0.029 (0.976)
Good sleep quality (poor sleep quality=reference)	0.052* (0.023)	7.090*** (<0.001)
No. of Leisure activity	0.092*** (<0.001)	2.224*** (<0.001)
Living alone	0.037* (0.044)	1.614 (0.062)
Friends connection	0.022 (0.115)	2.509*** (<0.001)
Family connection	0.012 (0.680)	−1.461 (0.285)
_cons	1.164*** (<0.001)	76.492*** (<0.001)
sigma		
_cons	0.218*** (<0.001)	
N	1278	1278

p-values in parentheses, * p < 0. 05, ** p < 0.01, *** p < 0.001

Age was negatively correlated with EQ-5D and EQ-VAS scores. While gender, educational level, and marital status were not significantly associated with both health utility scores.

Regarding diseases factors, the results showed that the number of drugs consumed per day, hearing and visual impairment was negative associated with EQ-5D scores. Visual impairment might exert greater impact on health utility than hearing impairment, as hearing impairment was not significantly associated with EQ-VAS score but visual impairment affected both health utility scores. Compared to respondents who did not have chronic diseases, respondents who had chronic diseases reported lower EQ-VAS scores by 1.6.

In terms of lifestyle factors, BMI was positively and significantly correlated to both health utility scores. A 1-unit increase in BMI would increase the EQ-VAS score by 0.16 (P<0.1). Drinking was negatively correlated with the EQ-5D score, while smoking was not significant in both regressions. Compared to the oldest-old respondents who self-reported having bad sleeping quality, the respondents who had good sleeping quality reported higher EQ-VAS score by 7.09 on average. If the respondent had one more type of leisure activity, his/her EQ-VAS score would increase by 2.22 points.

In terms of family and social support factors, respondents who live alone were more likely to report a higher VAS score by 1.61 than those living with other people. For those oldest-old respondents who had at least one friend to contact per month reported higher EQ-VAS score compared to those who had no friends to talk by 2.51 points. Social connection played a positive role in their self-reported health utility.

### Associated factors of each EQ-5D dimension

The regression results for the ordered probit models on each health dimension were reported in Table 4. The same set of explanatory variables were used in the regressions as Table 3. The coefficients from ordered probit regressions were not marginal effects, so the magnitude of the coefficients could not be explained as the marginal impact of the explanatory variable on the dependent variable. However, the sign of the coefficients represented the relationship between them. The higher the outcome variable (1, no problem; 2, moderate problem; 3, severe problem), the more severe the problem is. Thus, a positive sign represented a negative relationship between risk factors and outcome variables.

**Table 4:** Ordered Probit Regressions Analyses on EQ-5D Dimensions

	**Regression coefficients (p-value)**				
	**Mobility**	**Selfcare**	**Usual activity**	**Pain/discomfort**	**Depression/anxiety**
Age	0.032*** (<0.001)	0.024*** (<0.001)	0.033*** (<0.001)	0.008 (0.116)	−0.025*** (<0.001)
Male	−0.191 (0.063)	−0.051 (0.628)	−0.087 (0.403)	−0.159 (0.115)	−0.141 (0.321)
Illiterate	−0.257* (0.032)	−0.253 (0.060)	−0.205 (0.097)	0.117 (0.317)	−0.270 (0.081)
Married	−0.016 (0.965)	0.021 (0.961)	0.270 (0.518)	−0.330 (0.321)	−0.047 (0.909)
Widowed	0.482 (0.183)	0.425 (0.321)	0.660 (0.112)	−0.048 (0.884)	0.139 (0.733)
Chronic diseases					
(no comorbidity=reference)	0.008 (0.935)	0.065 (0.540)	0.062 (0.538)	0.003 (0.973)	−0.163 (0.226)
No. of drugs consumed per day	0.099* (0.046)	0.100* (0.039)	0.037 (0.421)	0.197*** (<0.001)	0.172** (0.005)
Hearing impairment	0.373*** (<0.001)	0.342** (0.001)	0.346** (0.001)	0.205 (0.056)	0.413** (0.001)
Visual impairment	0.310** (0.002)	0.201* (0.041)	0.177 (0.065)	0.100 (0.318)	0.214 (0.081)
BMI	−0.025* (0.021)	−0.006 (0.611)	−0.013 (0.241)	−0.011 (0.281)	0.001 (0.962)
Smoking	−0.169 (0.338)	−0.018 (0.914)	−0.118 (0.468)	−0.044 (0.775)	−0.122 (0.552)
Drinking	0.170 (0.140)	0.175 (0.108)	0.307** (0.005)	0.219* (0.048)	−0.026 (0.864)
Good sleep quality					
(poor sleep quality=reference)	−0.261 (0.060)	−0.008 (0.957)	−0.145 (0.257)	−0.342** (0.009)	−0.646*** (<0.001)
No. of Leisure activities	−0.507*** (<0.001)	−0.665*** (<0.001)	−0.528*** (<0.001)	−0.093* (0.034)	−0.338*** (<0.001)
Living alone	−0.233* (0.019)	−0.259* (0.017)	−0.221* (0.028)	−0.018 (0.851)	0.120 (0.348)
Friends connection	−0.108 (0.177)	0.021 (0.806)	−0.114 (0.148)	−0.111 (0.156)	<0.001 (0.996)
Family connection	0.059 (0.714)	0.012 (0.938)	−0.014 (0.928)	−0.039 (0.793)	−0.536** (0.002)
cut1					
_cons	1.845** (0.009)	2.175** (0.004)	2.496** (0.001)	0.500 (0.471)	−2.347** (0.009)
cut2					
_cons	4.361*** (<0.001)	4.114*** (<0.001)	4.802*** (<0.001)	3.089*** (<0.001)	−0.425 (0.639)
N	1278	1278	1278	1278	1278

p-values in parentheses, * p < 0. 05, ** p < 0.01, *** p < 0.001

Age was negatively correlated to mobility, self-care, usual activity, but positively correlated to depression and anxiety. We did not find significant results for gender, educational level, and marital status.

The respondents who consumed more drugs per day were more likely to report problems in mobility, self-care, pain/discomfort, and depression/anxiety. Similarly, hearing and visual impairment both negatively affected 5 health dimensions for the oldest-old respondents.

Among life-style factors, BMI was positively correlated to mobility. Those with higher BMI were more likely to report fewer problems in mobility. Those who drink alcohol were more inclined to report problems in dimensions of usual activity and pain/discomfort, but no significant association was found between smoking behaviour and any health dimensions. Respondents who had more types of leisure activities were less likely to report problems in every health dimensions. Compared to bad sleeping quality, both normal and good sleeping quality was positively associated with depression and anxiety. Those who reported good sleeping quality are more likely to have fewer problems in mobility and pain/discomfort.

When considering family and social support factors, whether the oldest old respondent was living alone was positively correlated to mobility, self-care, and usual activity, but not associated with pain/discomfort and depression/anxiety. It was reasonable because only those who did not have functional problems were capable of living alone. Family communication was positively affected depression/anxiety. Those who had at least one family member to contact per month were less likely to report problems in the dimension of depression/anxiety.

## Discussion

To the best of our knowledge, this was the first study to evaluate the health quality of life and its associated factors among oldest-old adults in China. EQ-5D instrument has been widely used in pharmacoeconomic analysis and the evaluation of health policy programme as a consistent and transparent measurement of the output of health care and public health system (22).

The explore of the health dimensions showed that moderate and severe problems were more frequent in the dimensions of mobility (50%) and usual activities (49%). Few problems were reported in the dimension of anxiety/depression (12%). The results of this study were consistent with the respective values for elders aged above 75 years old from six European countries (23). The percentages of reporting problems in any health dimensions in our sample (65%) were slightly lower than those from study on six European countries (66%), even when the respondents in our sample were 20 years older.

We found gender differences in answers to EQ-5D questions. Women had lower proportions of reporting no problems and higher proportions of reporting extreme and moderate problems regarding all 5 health dimensions. In general, self-reported health utility scores for women was worse than men. This gender difference was similar to other international studies that women reported more problems on every health dimensions, and had lower EQ-5D scores and EQ-VAS scores (16, 24, 25). In the estimation of associated factors, gender effects seemed to be washed out by age effect considering the male respondents were 5 years younger than females.

Advanced age was significantly associated with more problems in 4 health dimensions except for anxiety/depression. After reaching 90 years old, older age was associated with fewer problems in the dimension of anxiety/depression. This was in accordance with a recent study showing optimism and resistance is associated with exceptional longevity (85+) in 2 cohorts of the US population (26).

As China is aging rapidly, the government has made great efforts to provide institutional care and long-term care insurance in order to increase access to care and reduce the financial barriers for disabled older people (27). However, the long-term care system in China is still in the stage of infancy and the oldest-old population suffer more chronic conditions and physical and mental problems. Thus, more attentions should be paid to promote the quality of life for those oldest-old adults. Findings from this study suggested that quality of life was not only associated with age-related diseases, but also correlated with a range of lifestyle factors, and factors indicating social and family support. After controlling for demographic variables including age, gender, education and marital status, better health-related quality of life was significantly associated with higher BMI, no drinking habit, more leisure activities, living with family members, good sleeping quality, better social and family connections, fewer numbers of drugs consumed per day, without having hearing or visual impairment, and fewer chronic conditions. Under the circumstance of a rapidly aging society, it is essential to provide more educational services on lifestyle choices, healthy eating programmes, sleeping quality improvement programs, as well as smart community services to provide more social support especially for those living alone and having fewer leisure activities.

Results from previous research found a significant correlation between lower education and lower EQ-5D scores and EQ-VAS scores (23, 24), but the education level was not significant in all regressions in this study. It might be due to the small variation of the educational level in our sample. A vast majority was illiterate (83%), and even if they were literate, the educational level was quite low (primary school), bearing in mind that China was extremely poor at their school age. Smoking was a common risk factor of health-related quality of life in general population (28–30) and in younger elder adults (24, 31) , but it was not associated with any EQ-5D health dimensions among oldest-old population in this study. A few studies had found consistent results among oldest old population. For example, Cohen-Mansfield suggested that the adverse effect of tobacco consumption on mortality decreased or disappear among the population aged 85 and older, which was largely due to the survivorship bias (32).

There was limited literature that reported health-related quality of life and its determinants among Chinese oldest-old adults. This study filled the gap of the literature and implemented a variety of socio-demographic, lifestyle and social support variables into the analysis. In addition, the analysis has a relatively large sample size of the oldest old adults compared to similar international studies, which reduces the risk of reporting false-negative results and increases the robustness of the study. However, several limitations remained in the study and could be addressed. Firstly, although questions inquiring the income of households were contained in the questionnaire, the data quality of this variable did not fit for the analysis due to a large proportion of missing data and recall biases. We tried to substitute the income level with the sources of income (Supplement Table 2) and the results were consistent with our main findings. Secondly, only one wave of the data was available for the analysis, thus the causal inference between the associated factors and health-related variables was difficult to be identified. Future studies are in need to verify the casual relationship of certain variables to better inform the policy design. Thirdly, there is no specific EQ-5D norm for the oldest-old Chinese population, thus we adopted the TTO value set for the overall Chinese population. Further studies could be dedicated to develop the EQ-5D norm for this population to better understand their self-reported quality of life.

## Conclusion

In terms of self-rated health quality of life, mobility and usual activity were the two major problems for Chinese oldest-old. Besides, gender differences were observed in quality of life and female reported more problems in all health dimensions. The impairment of the health-related quality of life were associated with a range of socio-demographic factors, comorbidities, lifestyles, living arrangement, family support and social connections. These factors, which were modifiable to some extent, could be potentially targets on improving health quality of life among oldest old adults.

## Electronic supplementary material

Supplementary material, approximately 18.9 KB.

## Notes

*Ethical Standards:* All procedures performed in studies involving human participants were in accordance with the ethical standards of the institutional and/or national research committee and with the 1964 Helsinki declaration and its later amendments or comparable ethical standards. This article does not contain any studies with animals performed by any of the authors. Written informed consent was obtained from all men and women who participated in the CHCCS study. The Ethics Committee of the Hainan branch of the Chinese People’s Liberation Army General Hospital (Sanya, Hainan) approved the study protocol (No. 301hn11201601).
