# Supplementary material for: Health-Related Quality of Life and Associated Factors Among Oldest-Old in China
Source: J Nutr Health Aging. 2020 Jan 20;24(3):330–8. doi: 10.1007/s12603-020-1327-2 (PMC7064459; doi:10.1007/s12603-020-1327-2)
Supplement: Supplementary file 1 — Supplementary material, approximately 18.9 KB. [file 12603_2020_1327_MOESM1_ESM.docx]

**Supplement Table 1:** Chinese Time-Trade Off Value Set

|  | **Mobility** | **Self-care** | **Usual activity** | **Pain/discomfort** | **Depression/anxiety** |
| --- | --- | --- | --- | --- | --- |
| 1 | 0 | 0 | 0 | 0 | 0 |
| 2 | 0.099 | 0.105 | 0.074 | 0.092 | 0.086 |
| 3 | 0.246 | 0.208 | 0.193 | 0.236 | 0.205 |
| N_3_ | 0.022 | | | | |
| C | 0.039 | | | | |

**Supplement Table 2:** Regression Results on QALY and EQ-VAS Score

(with sources of income)

|  | (1) | (2) |
| --- | --- | --- |
|  | QALY | VAS |
| Age | -0.01^***^ | -0.17^***^ |
|  | (0.00) | (0.05) |
| Male | 0.02 | 1.18 |
|  | (0.02) | (0.90) |
| Illiterate | 0.02 | -0.85 |
|  | (0.02) | (1.05) |
| Married | 0.01 | 0.35 |
|  | (0.08) | (3.07) |
| Widowed | -0.09 | -0.16 |
|  | (0.08) | (3.05) |
| Bmi | 0.00^**^ | 0.14 |
|  | (0.00) | (0.10) |
| No. of drugs consumed per day | -0.03^***^ | -0.42 |
|  | (0.01) | (0.45) |
| Hearing impairment | -0.08^***^ | -0.66 |
|  | (0.02) | (0.97) |
| Visual impairment | -0.04^***^ | -2.36^***^ |
|  | (0.02) | (0.90) |
| Smoking | 0.04 | -0.39 |
|  | (0.03) | (1.33) |
| Drinking | -0.06^***^ | -0.08 |
|  | (0.02) | (0.98) |
| Types of leisure activities | 0.09^***^ | 2.22^***^ |
|  | (0.01) | (0.39) |
| Living along | 0.04^**^ | 1.56^*^ |
|  | (0.02) | (0.88) |
| Good sleeping quality | 0.05^**^ | 7.26^***^ |
| Ref=bad sleeping quality | (0.02) | (1.18) |
| Normal sleeping quality | 0.02 | 0.73 |
| Ref=bad sleeping quality | (0.02) | (1.15) |
| Friends communications | 0.03^*^ | 2.26^***^ |
|  | (0.01) | (0.71) |
| Family communications | 0.02 | -1.60 |
|  | (0.03) | (1.38) |
| Chronic diseases | -0.01 | -1.66^*^ |
|  | (0.02) | (0.93) |
| Income source: family members | -0.04 | 1.22 |
| Ref=pension | (0.03) | (1.33) |
| Income source: personal saving account | -0.13^***^ | 3.07 |
| Ref=pension | (0.05) | (3.44) |
| Income source: government | 0.01 | -0.33 |
| Ref=pension | (0.03) | (1.24) |
| Income source: others | 0.05 | -4.41 |
| Ref=pension | (0.06) | (2.95) |
| _cons | 1.18^***^ | 76.70^***^ |
|  | (0.14) | (6.31) |
| sigma |  |  |
| _cons | 0.22^***^ |  |
|  | (0.01) |  |
| *N* | 1245 | 1245 |

Standard errors in parentheses

^*^ *p* < 0.1, ^**^ *p* < 0.05, ^***^ *p* < 0.01
